# Hybrid Treatment of Complex Diseases of the Aortic Arch and Descending Thoracic Aorta by Frozen Elephant Trunk Technique

**DOI:** 10.3390/jcm12175693

**Published:** 2023-09-01

**Authors:** Jean Porterie, Aurélien Hostalrich, François Dagenais, Bertrand Marcheix, Xavier Chaufour, Jean-Baptiste Ricco

**Affiliations:** 1 Department of Cardiovascular Surgery, Centre Hospitalier Universitaire de Toulouse, 31300 Toulouse, France; marcheix.b@chu-toulouse.fr; 2 Department of Vascular Surgery, Centre Hospitalier Universitaire de Toulouse, 31300 Toulouse, France; hostalrich.a@chu-toulouse.fr (A.H.); chaufour.x@chu-toulouse.fr (X.C.); 3 Department of Cardiovascular Surgery, Institut Universitaire de Cardiologie et de Pneumologie de Québec, Québec, QC G1V 4G5, Canada; francois.dagenais@fmed.ulaval.ca; 4 Department of Vascular Surgery, Centre Hospitalier Universitaire de Poitiers, 86000 Poitiers, France; jean.baptiste.ricco@univ-poitiers.fr

**Keywords:** aneurysm, aortic arch, aortic dissection, frozen elephant trunk

## Abstract

The surgical management of acute and chronic complex diseases involving the aortic arch and the descending thoracic aorta remains challenging. Hybrid procedures associating total open arch replacement and stent-grafting of the proximal descending aorta were developed to allow a potential single-stage treatment, promote remodeling of the downstream aorta, and facilitate a potential second-stage thoracic endovascular aortic repair by providing an ideal landing zone. While these approaches initially used various homemade combinations of available conventional prostheses and stent-grafts, the so-called frozen elephant trunk technique emerged with the development of several custom-made hybrid prostheses. The aim of this study was to review the contemporary outcomes of this technique in the management of complex aortic diseases, with a special focus on procedural planning, organ protection and monitoring, refinements in surgical techniques, and long-term follow-up.

## 1. Introduction

The surgical management of acute and chronic complex diseases involving the aortic arch and the descending thoracic aorta (DTA) remains challenging. The conventional elephant trunk (ET) technique was introduced to facilitate a two-stage approach but carries significant mortality and morbidity due to the cumulative risk of two major procedures and a waiting period [1,2,3]. Otherwise, thoracic endovascular aortic repair (TEVAR) has greatly improved the outcomes of acute and chronic diseases of the DTA [4]. Nevertheless, the endovascular management of arch diseases is still associated with unresolved issues [5,6]. Hybrid procedures associating total arch replacement (TAR) and stent-grafting of the proximal DTA were developed to allow a potential single-stage treatment, promote the remodeling of the downstream aorta and facilitate a potential second-stage TEVAR by providing an ideal landing zone [7,8,9,10,11]. While these approaches initially used various homemade combinations of available conventional prostheses and stent-grafts, the so-called frozen elephant trunk (FET) technique emerged with the development of several custom-made hybrid prostheses [7,8,9,10,11]. According to current European and North American guidelines, TAR-FET should now be discussed for all pathologies involving the aortic arch [12,13,14]. The aim of this study was to review the contemporary outcomes of FET in the management of complex aortic diseases, with a special focus on procedural planning, organ protection and monitoring, refinements in surgical techniques, and long-term follow-up.

## 2. FET Technique: General Considerations

Contemporary studies of TAR-FET report in-hospital mortality rates ranging from 0% to 18.2%, which compares favorably with conventional techniques [8,9,10,11,15,16]. However, complications such as stroke, spinal cord injury (SCI), acute kidney injury, or bowel ischemia are devastating. However, these complications are correlated both to the underlying disease, the elective or urgent/emergent setting, and baseline patient conditions. For instance, TAR-FET has been shown to carry significantly higher perioperative morbidity and mortality as well as impaired long-term survival in older patients, whereas TAR-FET as a redo procedure after previous proximal and/or distal open or endovascular thoracic aortic repair is associated with acceptable outcomes in well-selected patients [17,18,19,20]. Thus, the reduction of morbidity and mortality to a minimum remains a major concern for the aortic teams and makes patient selection and perioperative management, including organ protection and monitoring, paramount.

### 2.1. Stroke

Current series on TAR-FET report stroke rates ranging from 0% to 20% [8,9,10,11,12,13,14,15]. Axillary artery cannulation and antegrade cerebral perfusion (ACP) are widely considered the safest option for brain protection while allowing moderate hypothermic circulatory arrest (MHCA ≥ 28 °C) rather than deeper hypothermia and are recommended in the current guidelines [13,14,21,22,23,24]. Unilateral ACP has previously been proven to be as effective as bilateral ACP while potentially decreasing injuries to the left common carotid artery (LCCA) and iatrogenic embolism [25]. However, bilateral ACP may be more effective in the case of circulatory arrest (CA) ≥ 50 min [26]. Moreover, cannulation of the LCCA and bilateral ACP should be performed when inadequate regional oxygenation in the left cerebral hemisphere under unilateral ACP is suspected. Thus, the accurate stratification of different risk-profile patients is critical. Preoperative computed tomography angiography (CTA) should include both the supra-aortic trunks and the circle of Willis [13], transcranial Doppler being a potential adjunct to assess the functionality of the circle of Willis but is more complex with regard to the intraoperative setup [27]. Bifrontal near-infrared spectroscopy (NIRS) allows to continuously monitor the balance of oxygen supply and demand in superficial cortical regions of the brain [28,29].

### 2.2. Spinal Cord Injury (SCI)

TAR-FET has been associated with higher SCI rates (up to 20%) than classic ET and other conventional aortic arch procedures, partly due to a faster post-operative false lumen (FL) or peri-graft space thrombosis [8,9,10,11,12,13,14,15]. A recent meta-analysis of 35 studies, including more than 3000 patients, reported a pooled SCI rate of 4.7% [15]. It is now well admitted that SCI is multifactorial, including perioperative hypotension, previous abdominal aortic aneurysm repair, and loss of left subclavian artery (LSA) inflow; thus, both its prevention and treatment should be integrated into a multimodal approach [13,30,31]. In the setting of open arch surgery, the data supporting systematic prophylactic cerebrospinal fluid (CSF) pressure monitoring and drainage remain scarce, and imaging is still not able to provide a reliable description of spinal cord vascularization [30,32,33]. When required, CSF drainage should target CSF pressure of 10–12 mmHg (prevention) or 8–10 mmHg (treatment) and be performed slowly (volume flow ≤ 40 mL/4 h) to prevent intracranial hypotension and consecutive brain damage [13,30]. Meanwhile, fast-track management of SCI with CSF drainage and adjunctive measures such as mean arterial pressure elevation (target above 80 mmHg) and hemoglobin level optimization (>10 mg/dL) are mandatory [13,30].

### 2.3. Long-Term Outcomes

The TAR-FET procedure can achieve a true single-stage treatment of aortic diseases limited to the level of the distal landing zone and promote remodeling of the DTA [8,9,10,11]. Nevertheless, a substantial subset of patients will require reinterventions on the DTA [34,35]. In a recent meta-analysis over 37 studies including 4178 patients, overall survival at 1, 3, and 5 years was 89.6%, 85.2%, and 82.0%, respectively, with freedom from reintervention of 93.9%, 89.3%, and 86.8%, respectively [11]. Thus, close follow-up in dedicated aortic centers is mandatory to decrease the risk of aortic-related events and to plan when needed a second-stage procedure [8,9,10,11,12,13,34,36].

**Figure 1:**
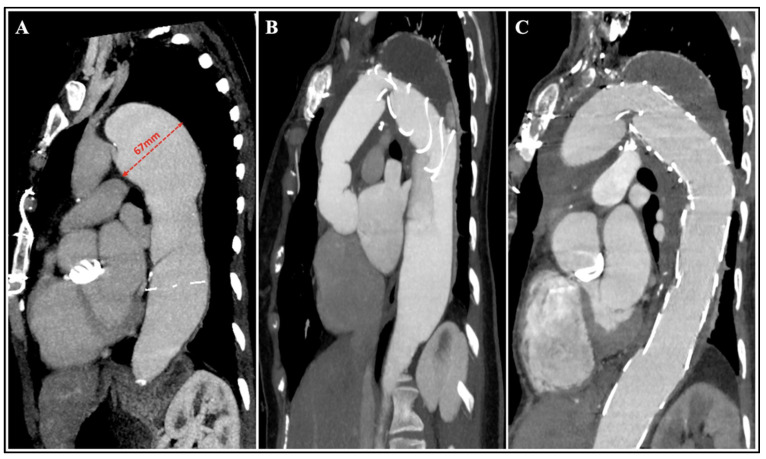
Multi-stage approach. CTA follow-up of a 68-year-old male patient with a complex aneurysm of the descending aorta involving the aortic arch, which developed 15 years after a previous mechanical Bentall procedure for aortic root aneurysm (**A**). The patient underwent redo TAR-FET as a first-step procedure (**B**), followed by second-stage TEVAR completion 4 months thereafter (**C**), allowing total exclusion and thrombosis of the aneurysmal sac without endoleak. CTA: computed tomography angiography; FET: frozen elephant trunk; TAR: total arch replacement; TEVAR: thoracic endovascular aortic repair.

In multilevel thoracoabdominal aortic diseases without an adequate proximal landing zone for TEVAR, a multi-step approach including TAR-FET as a first-stage procedure offering an ideal landing zone for subsequent TEVAR completion (Figure 1) has been associated with excellent clinical outcomes [37]. The isthmic circumferential suture of the FET eliminates the well-known drawbacks of TEVAR, such as proximal endoleak or stent-graft migration. On the other hand, the endoluminal sealing of the surgical suture line by the stent-graft improves hemostasis [38]. Distal fenestrated (F)/branched (B)-EVAR after prior TAR-FET has been associated with high technical success and favorable midterm survival, despite the risk of SCI as well as the requirement for reintervention are not low [39]. This feature is pivotal since secondary open repair is usually challenging and associated with higher mortality than endovascular completion [40]. However, when an open repair is required, the FET is also able to be clamped and shifts the docking place to at least a mid-thoracic level, with easier surgical access, reducing CA time and preventing laryngeal nerve injury. 

## 3. Aortic Dissection

### 3.1. Acute Aortic Dissection

In acute aortic dissection (AAD), TAR-FET has been pointed out to be able to resolve or prevent associated malperfusion syndrome by reopening the true lumen (TL) and depressurization of the FL [41,42,43]. However, pre-existing malperfusion syndromes have been associated with higher mortality after the procedure [44]. In the setting of type A AAD, TAR-FET can be performed with acceptable perioperative outcomes as compared with classical limited repairs in centers with expertise and adequate patient selection [9,45]. A recent meta-analysis of 17 studies, including 1300 patients undergoing TAR-FET for AAD, reported an average in-hospital mortality of 7.8%, ranging from 0% to 18.2% [46]. 

**Figure 2:**
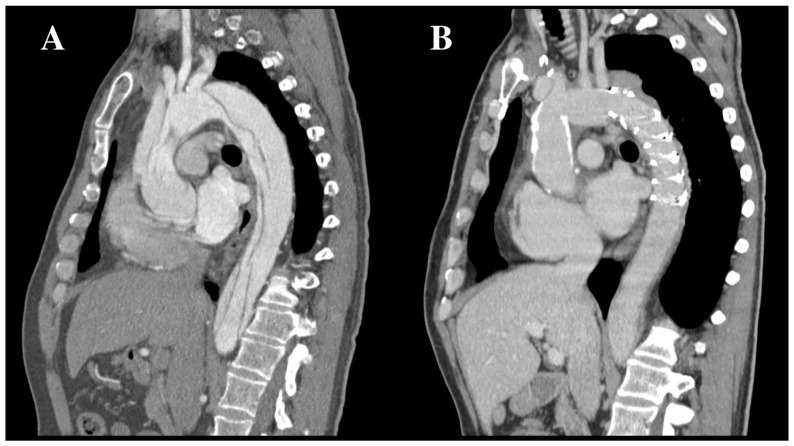
Positive aortic remodeling after frozen elephant trunk procedure. Preoperative (**A**) and 3-months post-operative (**B**) CTA of a 56-year-old male patient who experienced type A AAD and underwent emergent TAR-FET. Follow-up CTA highlighted the beneficial effect of FET on the shrinkage of the FL in the downstream aorta. AAD: acute aortic dissection; CTA: computed tomography angiography; FET: frozen elephant trunk; FL: false lumen; TAR: total arch replacement.

In AAD, TAR-FET decreases the risk of pseudoaneurysm at the level of the distal anastomosis by combining surgical sutures and endovascular sealing, excludes antegrade FL perfusion, stabilizes the dissecting membrane and expands the TL, favoring progressive FL thrombosis and shrinkage and participating in positive aortic remodeling (Figure 2) [9,47,48,49,50,51,52]. In a recent meta-analysis including 1295 patients undergoing TAR-FET for type A AAD, 1- and 5-year survival ranged from 80% to 100% and 68% to 96%, respectively, 1-year freedom from aortic re-operation was 86.5% to 100% and pooled average aortic remodeling (partial or complete FL thrombosis around the stent-graft) was 92%, to compare with those reported by the largest registries for conservative management ranging from 33.3% to 77.8% [46]. However, Berger et al. reported distal aortic failure (distal aortic reintervention, aortic diameter dilatation, and/or aortic-related death) in nearly half of a cohort of 186 patients undergoing TAR-FET for aortic dissections (AD), especially in acute cases, more extensive disease, and larger preoperative aortic diameters [36]. 

### 3.2. Chronic Aortic Dissection

After previous type A limited repair, one-third of patients have a residual dissection in the aortic arch, leading to aortic growth, also favored by re-entries at the distal anastomosis and antegrade FL perfusion. Most of these “type B” chronic aortic dissection (CAD), after previous type A repair, require secondary reinterventions. In this setting, redoing TAR-FET has been associated with low perioperative morbidity, positive aortic remodeling, and satisfying reoperation-free survival, especially in Marfan patients [18,19,53]. Otherwise, TAR-FET has demonstrated excellent results in patients with type B CAD and unsuitable anatomy for TEVAR, excluding the risks of retrograde dissection, type Ia endoleak, and possible stent-graft migration [54,55]. In CAD, a recent study pointed out post-operative larger aortic diameters and FL thrombosis at the DTA level as predictive factors of the development of malperfusion after the FET procedure, whereas the involvement of aortic branches by the dissection was not [56]. Risk factors for distal aortic reintervention, aortic diameter dilatation, and/or aortic-related death after TAR-FET for CAD are comparable to those observed in AAD, including more extensive disease, aortic diameter and the number of distal re-entries [36]. 

## 4. Surgical Refinements

### 4.1. Procedural Planning and Stent-Graft Selection

Several custom-made hybrid devices with various diameters, lengths, and structural characteristics are now available (Table 1). CTA is recommended as the first-line imaging modality, and post-processing enables multiplanar reformation to enhance measurements of aortic lengths and diameters [4,13]. On the basis of preoperative CTA, the endograft diameter should be determined to fit the intended landing zone, according to the underlying aortic disease: 5–15% oversizing in aneurysms, based on the aortic diameter at this level; no oversizing in AAD and/or connective diseases; and variable in CAD, where the sizing should tend to the mean TL diameter. Moreover, FET sizing should be planned in view of potential second-stage TEVAR.

Many authors recommend the use of short endografts, especially in acute cases, since the implantation of longer stent-grafts (≥150 mm) and extensive coverage of the intercostal arteries (beyond T7) has been shown to increase SCI risk [15,30,31]. On the other hand, Tan et al. hypothesized that the ‘‘cutoff’’ phenomenon could be involved in AD, where a distance ≥ 30 mm from the distal end of the stent-graft to the first untreated intimal tear would be associated with higher rates of paraplegia [57]. Otherwise, decreased FL thrombosis, negative aortic remodeling, and subsequently higher rates of aortic reinterventions are associated with shorter length of covered DTA (especially above T4-5), condition favored by the use of short (≤100 mm) stent-grafts and the “proximalization” (above zone 2) of the distal anastomosis [58,59]. Thus, preoperative planning based on 3D CTA is essential for an optimal tailor-made strategy, taking into consideration whether to achieve a primary distal seal or prepare a secondary completion and how to minimize the potential SCI risk. CTA planning is also pivotal in AD, where post-operative malperfusion should, in part, be prevented by careful evaluation of the extent of the intimomedial flap, the relationship between TL and FL, the locations of entry tears, and the origin of the branch vessels [12,13,14].

### 4.2. Stent-Graft Deployment

The use of a retrogradely inserted guidewire (preferably via the femoral artery) under angiographic or transesophageal echocardiography control may be helpful to secure FET deployment within the TL in AD or to facilitate its guidance over thrombi in aneurysms. Angioscopy may be an additional intraoperative tool in visualizing the landing zone and endoluminal obstacles [60]. Inadvertent landing of the FET into the FL can be caused by an unnoticed distal re-entry tear or the occurrence of a distal stent-graft-induced new entry (dSINE) and lead to pressurization of the vulnerable FL and narrowing of the TL with potential subsequent malperfusion syndromes and negative remodeling of the DTA. In such cases, controlled fenestration of the dissection flap (either open or endovascular approach), possibly followed by second-stage TEVAR, has been proposed [61,62]. 

### 4.3. Distal Anastomosis

The “proximalization” of FET fixation from zone 3 (aortic isthmus) to zone 2 (LSA) or above has been proposed by several teams in order to facilitate the distal anastomosis as well as bleeding control, to reduce the duration of lower-body MHCA, decrease segmental coverage and SCI risk, as well as prevent left laryngeal nerve injury [63,64,65]. A recent meta-analysis of 64 studies, including 7967 patients, underscored that the distal anastomosis in zone 2 was associated with a lower rate of renal failure compared with zone 3 [66]. Moreover, combining zone 2 FET with LSA debranching further minimizes the duration of the arch repair and allows the perfusion of all three arch vessels for additional cerebral and spinal cord protection [67]. Ultimately, a new sutureless device has been proposed to simplify TAR-FET procedures in a recent pilot study of 10 patients, with encouraging outcomes in terms of shortened MHCA and midterm follow-up, despite further evaluation being mandatory [68]. 

### 4.4. Neck Vessels and Management of the Left Subclavian Artery

Supra-aortic trunks reimplantation can be time-consuming, and the LSA may be especially challenging due to its deep position. Thus, various refined techniques have been developed to make revascularization of the neck vessels easier, TAR faster, and CA shorter, with encouraging outcomes. Despite LSA occlusion and subsequent extra-anatomical revascularization may be performed in patients with challenging anatomies, a predictive model able to reliably discriminate higher-risk from low-risk situations is still lacking. Moreover, the collateral network and the 4-territory concepts have led to advocate that preservation of LSA should participate in preventing stroke (cerebellar perfusion) and SCI (upper inflow into the anterior spinal artery), especially in the setting of extensive segmental coverage, urgent/emergent procedure, and/or prior aortic interventions [13,15,30,32,33]. Several authors have proposed LSA ligation and extra-anatomic bypass with a side arm from the aortic graft through the first intercostal space to the left axillary artery, allowing multisite (including LSA) perfusion techniques in order to improve cerebral and medullar protection during arch reconstruction [67,69]. In elective cases, other authors have proposed to perform a LCCA-LSA bypass associated with plugging of the prevertebral LSA (“debranch-first” technique) prior to the TAR-FET procedure [70]. Grabenwöger et al. demonstrated the feasibility of implanting a newly designed FET prosthesis with a stented side branch to the LSA combined with distal anastomosis into zones 1–2 [71]. Other teams have proposed a branched/fenestrated FET technique or modified in situ reimplantation of the supra-aortic trunks [72,73,74]. Ultimately, first-in-man experience has recently been reported with the 3-zone E-Novia stent-graft (JOTEC/CryoLife GmbH, Hechingen, Germany) combining: (i) a proximal polyester portion for zone 0 anastomosis; (ii) a nitinol-braided, uncovered stent for arch coverage, and (iii) a distal covered z-shaped nitinol stent (Table 1) [75].

### 4.5. Lower-Body Perfusion

Several studies emphasized that lower-body MHCA duration negatively impacts visceral risk despite the theoretical protective effect of hypothermia [76,77]. In order to reduce lower-body MHCA time and improve distal organ protection, several authors advocated for prompt lower-body reperfusion after completion of the distal anastomosis, using a side branch or a balloon cannula as an endoclamp within the FET [78,79,80]. The Ancona team and other authors have proposed off-pump retrograde vascular stent-graft deployment in distal arch/DTA and the use of a retrograde endoballoon for prompt lower-body reperfusion, allowing TAR during uninterrupted normothermic cerebral and lower-body perfusion [81]. 

### 4.6. Heart Protection

While cold blood cardioplegia in an antegrade as well as retrograde fashion is effective and widely used, selective continuous normothermic myocardial perfusion during arch repair (“heart beating” concept) has been proposed in selected patients to reduce cardioplegic arrest times and myocardial injury, improve cardiac recovery, and allow more extensive procedures [82]. However, this technique requires cross-clamping to be safely feasible, either due to the unaffected native ascending aorta or ascending prosthesis. Appropriate unloading is also pivotal for myocardial protection.

### 4.7. How to Do It: Step-by-Step Toulouse Technique

Intraoperative monitoring involves: (i) left radial and femoral arterial pressures; (ii) central naso-pharyngeal, bladder, and rectal temperatures; and (iii) cerebral oxygenation through NIRS. A cardio-pulmonary bypass (CPB) is conducted between the right atrium and the right axillary artery unless unfavorable conditions (e.g., hemodynamic instability, dissected, or Lusoria right subclavian artery) preclude the cannulation of this vessel and lead to the femoral approach. The left ventricle is unloaded by a vent through the right superior pulmonary vein, and myocardial protection involves retrograde infusion of cold blood cardioplegia.

Once moderate hypothermia is achieved (24–28 °C at the bladder probe), MHCA and unilateral ACP through the right axillary artery (flow rate 10 mL/kg/min after clamping of the supra-aortic vessels) are initiated. The ascending aorta and aortic arch are resected. In case of unsatisfying backflow in the LCCA and/or inappropriate oximetry in the left cerebral hemisphere, an additional cannula is introduced into the LCCA for bilateral ACP. The cerebral perfusion is kept 2 °C lower than systemic MHCA. The distal anastomosis is prepared at zone 2 or 3 by circumferential placement of interrupted 2-0 polyester sutures with PTFE pledgets on the intimal side. 

**Figure 3:**
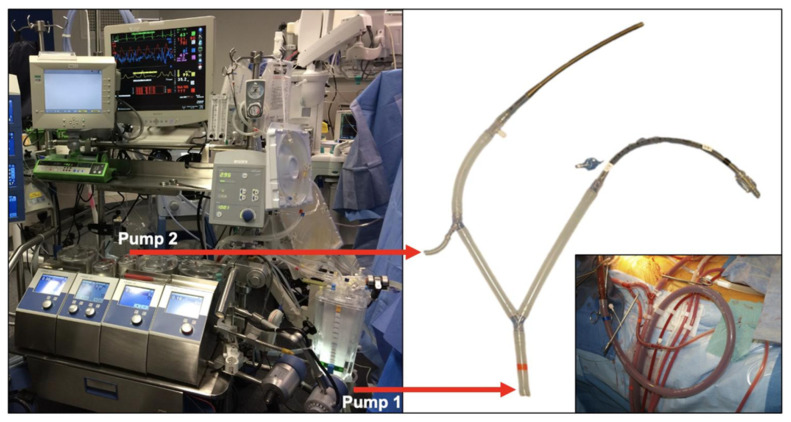
Modified cardiopulmonary bypass circuit. Arterial tubing setup with dual pump system.

**Figure 4:**
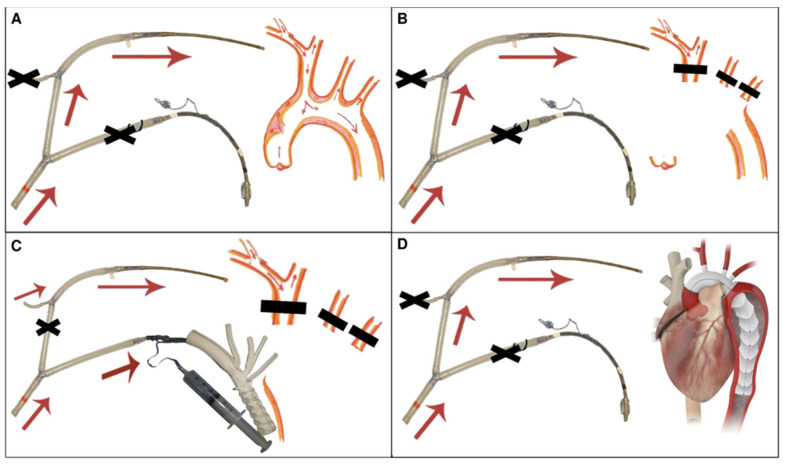
Operative steps for cerebral and thoraco-abdominal perfusions during MHCA. (**A**) Mode 1: systemic perfusion and cooling performed by pump 1. (**B**) Mode 1: supra-aortic trunks clamped, ACP performed by pump 1, and lower-body MHCA. Resection of the aortic arch. (**C**) Mode 2: ACP performed by pump 2 and thoraco-abdominal perfusion by pump 1, through a balloon canula deployed in the the FET stent-graft. (**D**) Mode 1: rewarming, proximal repair, and CPB weaning. ACP: antegrade cerebral perfusion; CPB: cardio-pulmonary bypass; FET: frozen elephant trunk; MHCA: moderate hypothermic circulatory arrest.

Our team exclusively uses the Thoraflex Hybrid prosthesis (Terumo Aortic, Inchinnan, UK). After the careful antegrade introduction of the device into the proximal DTA, the sheath is removed, the endoprosthesis deployed, and the distal anastomosis completed. Then, each arch vessel is sequentially anastomosed to the dedicated prosthetic branch in an end-to-end fashion. In case of unfavorable anatomical conditions or prior debranching, the LSA might be excluded. Whereas all these steps were initially performed during MHCA without distal perfusion, we subsequently modified our technique to perform antegrade thoracoabdominal perfusion after completion of the distal anastomosis (Figure 3 and Figure 4, Video S1).

Once the arch reconstruction is completed, general CPB is resumed after de-airing, and the proximal concomitant procedures are performed during rewarming. Finally, the proximal end of the arch graft is anastomosed either to the native ascending aorta or the proximal graft. After de-airing of the heart, the clamp is removed, and CPB is progressively weaned once rewarming is completed.

### 4.8. Perioperative Imaging

Ideally, performing such procedures in a hybrid room with imaging facilities is a valuable adjunct to allow prompt evaluation and ad hoc endovascular treatment of malperfusion or thoracic aortic rupture. Likewise, early post-operative CTA evaluation should be performed to confirm the absence of complications after the procedure.

## 5. Follow-Up

### 5.1. Follow-Up Imaging

All patients are followed in outpatient clinics and undergo iterative CTA or magnetic resonance angiography, from the neck vessels to the ilio-femoral arteries, ideally before discharge and regularly thereafter. In AAD and CAD, FL patency, as well as TL, FL, and total aortic diameters, should be assessed at each aortic level. In aneurysms, endoleaks should be assessed according to recommendations for TEVAR [83]. 

### 5.2. Distal Stent-Graft-Induced New Entry

dSINE may develop at any time after the FET procedure, with similar rates in AAD and CAD, and favor FL patency and aortic dilatation [84]. Mechanical stress of the flap on the distal edge of the stent-graft caused by retrograde FL expansion in the transition zone between a stent-covered and non-covered aorta could be a likely mechanism of dSINE [85]. The risk of dSINE should be decreased by avoiding oversizing in AAD as well as CAD and by selecting a shorter stent facilitating the direct control of a frail distal landing zone [84]. Other authors have proposed the coverage of the distal stent edge using a conventional graft reducing radial force and mechanical stress on the intima [86]. Finally, such dSINE are usually amenable to TEVAR completion with satisfying outcomes [87].

### 5.3. Stent-Graft Kinking

Only a few cases have been reported in a recent review, but graft kinking is another critical complication, with multiple theories regarding its mechanism, including the direction of blood flow and the length and position of the stent-graft in relation to the aortic curvature [88]. Balloon dilatation, TEVAR, or open repair have been proposed as possible management strategies.

### 5.4. Infections

FET infections are rare but challenging situations without specific guidelines regarding management. As in all vascular graft infections, the gold-standard strategy in patients deemed to be suitable for a redo procedure remains the removal of the prosthesis, followed by anatomical or extra-anatomical reconstruction, with silver-coated vascular grafts or alternative substitutes such as homograft or bovine tubular grafting [89]. Alternatively, conservative management with long-term suspensive antibiotic therapy could be an option in frail patients with a prohibitive risk for surgery. 

## 6. Alternative Techniques

In contrast to DTA, where TEVAR has become the standard of care, the ascending aorta is characterized by high shear stresses, four-dimensional movements during cardiac cycles, as well as the proximity of the coronary ostia and aortic valve. Endovascular arch repair requires a favorable proximal landing zone: (i) diameter ≤ 38 mm; (ii) length ≥ 25–30 mm (inner curvature); (iii) angulation > 60°; and (iv) no calcification or thrombus; as well as at least one adequate access vessel [13]. Moreover, TEVAR is contraindicated in patients affected by connective tissue disease and in those deemed to be at high risk of retrograde dissection. 

### 6.1. Transposition (Debranching) of Supra-Aortic Vessels and TEVAR

Despite the hybrid approach avoids CPB, aortic cross-clamping and CA, with potential benefits in higher-risk patients, evidence of its superiority over open TAR-FET is lacking [90]. Moreover, given manipulations during debranching as well as TEVAR, hybrid arch repair has been associated with significant rates of cerebrovascular events [90,91]. In the setting of type A AAD, hybrid repair could achieve favorable outcomes; nevertheless, it requires ascending aorta replacement in order for the prosthetic graft to provide a suitable proximal landing zone for the endograft [92].

### 6.2. Fenestrated and Branched Endovascular Arch Repair

The development of F-EVAR and B-EVAR aimed at mitigating the risks associated with open TAR and a recent meta-analysis of over 693 patients have shown in-hospital mortality and stroke rates as low as 2.5% and 4.8%, respectively, given appropriate patient selection [93]. However, endoleaks and reinterventions rates are not low [5,6,93]. Finally, provided careful patient evaluation and an expert team, these techniques are an option for patients deemed to be unsuitable or at prohibitive risk for open TAR. However, the devices currently require a manufacturing time (4 to 8 weeks), precluding their use in emergent situations. 

### 6.3. Other Options

Parallel grafts (PG), including chimney and periscope grafts as well as the sandwich technique, are bare or covered stents deployed in one or more supra-aortic vessels parallel to the main aortic arch stent-graft. The main advantages of PG techniques over F/B-EVAR are that they are off-the-shelf available. However, due to the gutters between grafts, these techniques carry a high risk of type I endoleak, raising the risk of aneurysm rupture. Data on aortic arch PG are scarce, with 30-day mortality ranging between 0 and 29% and early type I endoleak between 0 and 44% [94]. Data on long-term follow-up are still lacking; thus, PG techniques may be considered in the urgent/emergent setting for patients deemed inoperable or as a bail-out strategy in case of unintended covering of a supra-aortic vessel. 

## 7. Multidisciplinary Teams and Aortic Centers

Multidisciplinary aortic teams should be closely involved from diagnosis and treatment to follow-up. Indeed, patient selection and assessment are pivotal to selecting the best therapeutic strategy and achieving optimal outcomes. Additionally, centralization of care of aortic arch pathologies in large centers should be promoted to provide each patient with an entire range of treatment options with the availability of a hybrid operative room. Finally, a structured follow-up of all patients is pivotal to controlling the quality of repair and the potential evolution in non-treated aortic segments [13].

## 8. Gaps in Evidence and Future Perspectives

The population requiring aortic arch procedures is small compared to other cardiovascular populations, the caseload is low in many centers, and there is heterogeneity in therapeutic approaches. As a result, gaps in evidence still remain, including: (i) reliable surgical risk scoring systems; (ii) selection of the optimal approach in aortic arch diseases, especially the extent of index surgery in type A AAD; (iii) a better understanding of pathophysiology and optimal prevention of perioperative stroke, SCI, and other organ dysfunctions; (iv) standardization of follow-up after procedure; and (v) effects of caseloads and centralization of care on outcomes. In this regard, the development of prospectively maintained, large multicentric clinical databases for aortic arch pathologies should be promoted. 

## 9. Conclusions

TAR-FET is safe, effective, and reliable for obtaining primary aortic repair in a single stage in well-selected patients. Otherwise, TAR-FET favors FL thrombosis and aortic remodeling in AD and facilitates any secondary intervention, providing an ideal landing zone for TEVAR completion. Selection and patient management by multidisciplinary aortic teams, accurate preoperative assessment of the aortic anatomy, reliable methods of organ protection and monitoring, refinements in surgical techniques, and strict follow-up protocol should result in improved outcomes. 

## Figures and Tables

**Table 1:** FET devices. Several custom-made hybrid devices with various diameters, lengths, and structural characteristics are available. FET: frozen elephant trunk.

Current Custom-Made Hybrid Devices for Fet Procedures							
**Devices**	**E-vita^™^ Open & Open Plus** Jotec GmBH, Hechingen, Germany	**E-vita^™^ Open Neo** Jotec GmBH, Hechingen, Germany	**E-Novia** Jotec/CryoLife GmbH, Hechingen, Germany	**Thoraflex^™^ Hybrid prosthesis** Terumo Aortic, Inchinnan, Scotland, UK	**Cronus** MircoPort, Shanghai, China	**J Graft Frozenix** Japan Lifeline Co., Tokyo, Japan	**Cook hybrid stent-graft** Cook, Bloomington, IL
	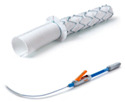	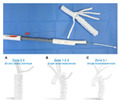	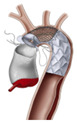	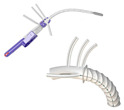	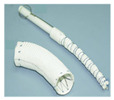	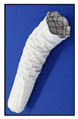	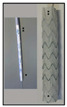
**General considerations**	The E-vita^™^ Open Plus is an updated iteration of the E-vita^™^ Open, obviating the need for pre-sealing with fibrin glue.	Double stent at the distal end for more apposition to the aortic wall. Revised delivery system, shorter and more ergonomic.	3-zone stent-graft with an intermediate nitinol-braided, noncovered stent for arch coverage.	Proximal unstented graft and distal self-expanding endograft with an intermediate collar.	Extra centimeter of Dacron sewing cuff at the proximal and distal ends.	Unique interconnected double-layered oval-shaped nitinol stents, which may be the best design for conforming to the aortic curvature.	Available in standard off-the-shelf sizes for emergent cases; can also be customized for elective cases.
**Stent-graft** **Diameter** **Lengths**	Z-shaped nitinol 24–40 mm 13–16 cm	Z-shaped nitinol 20–40 mm 12–13 cm/17–18 cm	Z-shaped nitinol 24–36 mm 10 cm	Saddle-shaped nitinol 24–40 mm 10 cm/15 cm	Z-shaped conichrome 21–32 mm 4–15 cm	Oval-shaped nitinol 21–39 mm 6–12 cm	Z-shaped nitinol
**Arch graft** **Diameter** **Length**	Combined 24–40 mm 5 or 7 cm	Combined 26, 28 and 30 mm 10 cm	Nitinol noncovered stent	Combined 22–32 mm 15 cm	Separated	Combined 21–39 mm	Separated
**Distal anastomosis** **Zone**	2–3	3 configurations: Straight: zone 2–3; Branched: zone 1–3; Trifurcated: zone 0–1.	0	2 configurations: Straight (Ante-Flo): zone 2–3; Branched (Plexus): zone 0–3.	0–3	2–3	0–3
**Sewing collar**	Open Plus	Yes	Yes	Yes	Yes	No	Yes
**Neck vessels reimplantation**	«En-bloc» technique	Straight: en-bloc; Branched/trifurcated: end-to-end.	NA	Straight: en-bloc; Branched: end-to-end.	En-bloc/End-to-end	En-bloc	En-bloc/End-to-end
**Perfusion branch**	No	Yes	No	Yes	No	No	No

## Data Availability

Not applicable.

## Supplementary Materials

The following supporting information can be downloaded at: https://www.mdpi.com/article/10.3390/jcm12175693/s1, Video S1: How to do it: step-by-step FET procedure (Toulouse university hospital).
